# Whole Genome Sequencing and Analysis of Chlorimuron-Ethyl Degrading Bacteria *Klebsiella pneumoniae* 2N3

**DOI:** 10.3390/ijms20123053

**Published:** 2019-06-22

**Authors:** Cheng Zhang, Qingkai Hao, Zhengyi Zhang, Xianghui Zhang, Hongyu Pan, Jiahuan Zhang, Hao Zhang, Fengjie Sun

**Affiliations:** 1 College of Resource and Environment, Jilin Agricultural University, Changchun 130118, China; zc15568039699@163.com (C.Z.); godrichao@163.com (Q.H.); 3330100@163.com (Z.Z.); zhjh63@126.com (J.Z.); 2 College of Plant Sciences, Jilin University, Changchun 130062, China; zhangxianghui1982@126.com (X.Z.); panhongyu@jli.edu.cn (H.P.); 3 School of Science and Technology, Georgia Gwinnett College, Lawrenceville, GA 30043, USA

**Keywords:** *Klebsiella pneumoniae* 2N3, chlorimuron-ethyl degrading bacteria, genome, third generation of high-throughput DNA sequencing

## Abstract

*Klebsiella pneumoniae* 2N3 is a strain of gram-negative bacteria that can degrade chlorimuron-ethyl and grow with chlorimuron-ethyl as the sole nitrogen source. The complete genome of *Klebsiella pneumoniae* 2N3 was sequenced using third generation high-throughput DNA sequencing technology. The genomic size of strain 2N3 was 5.32 Mb with a GC content of 57.33% and a total of 5156 coding genes and 112 non-coding RNAs predicted. Two hydrolases expressed by open reading frames (ORFs) 0934 and 0492 were predicted and experimentally confirmed by gene knockout to be involved in the degradation of chlorimuron-ethyl. Strains of ΔORF 0934, ΔORF 0492, and wild type (WT) reached their highest growth rates after 8–10 hours in incubation. The degradation rates of chlorimuron-ethyl by both ΔORF 0934 and ΔORF 0492 decreased in comparison to the WT during the first 8 hours in culture by 25.60% and 24.74%, respectively, while strains ΔORF 0934, ΔORF 0492, and the WT reached the highest degradation rates of chlorimuron-ethyl in 36 hours of 74.56%, 90.53%, and 95.06%, respectively. This study provides scientific evidence to support the application of *Klebsiella pneumoniae* 2N3 in bioremediation to control environmental pollution.

## 1. Introduction

Chlorimuron-ethyl, also known as chlorsulfuron-ethyl, is a type of selective pre- and post-emergence herbicide, which is widely used for the control of broad-leaved weeds in soybean fields [1]. Although chlorimuron-methyl is a herbicide with high efficiency and low toxicity, it has a long-lasting effect and is phytotoxic to many types of sensitive crops such as corn, sorghum, rape, melon, potato, and beet, thus limiting the use of this herbicide [1]. The degradation of chlorimuron-ethyl in the natural environment is accomplished mainly by chemical hydrolysis and microbial degradation. Compared with chemical hydrolysis, microbial degradations of environmental pollutants have been widely studied because of their high efficiency and safety. Bacteria in the genus *Klebsiella* are widespread in nature with several strains shown to be suitable for carrying out bioremediation due to their fast growth, a thick capsule on the surface of the cell wall, and resistance to pollutants [2,3]. For example, *Klebsiella* sp. CPK degrades the insecticide chlorpyrifos [4], *Klebsiella oxytoca* degrades cypermethrin [5], *Klebsiella* sp. NIII2 is able to produce glycoprotein flocculant [6], and *Klebsiella pneumoniae* J1 displays specific adsorption of Sulfamethoxazole [7,8,9]. Furthermore, *K. pneumoniae* 2N3, a gram-negative bacterium in the family of Enterobacteriaceae, can degrade not only chlorimuron but also other sulfonylurea herbicides such as metsulfuron-methyl, tribenuron-methyl, rimsulfuron, ethametsulfuron, and nicosulfuron [10]. A recent study has proposed the pathway for the degradation of chlorimuron-ethyl by another species in the Enterobateriaceae family, *Enterobacter ludwigii* sp. CE-1 [11]. Specifically, the sulfonylurea bridge in chlorimuron-ethyl is first cleaved, and then the intermediate products are converted to saccharin by hydrolysis and amidation. This biodegradation process is similar to those revealed in many other microorganisms that degrade chlorimuron-ethyl [12].

Microbial degradation of pesticide residues is mainly dependent on the production of the corresponding degradation enzymes. To date, many studies on the biodegradation mechanisms of sulfonylurea herbicides have focused on the degradation products and the metabolic pathways of compounds in pesticides [13,14]. However, there are only a few reports on the degradation enzymes produced by microorganisms. It has been demonstrated that two cytochrome P450 monooxygenases (P-450SU1 and P-450SU2) from *Streptomyces griseolus* are involved in the oxidation of sulfonylureas [15,16]. The nicosulfuron-degrading protein flavin monooxygenase (FMO) from *Talaromyces flavus* LZM1 was isolated and found to mainly degrade nicosulfuron by breaking the urea bridge [17]. Esterase SulE was obtained from *Hansschlegelia zhihuaiae* S113 and was reported to generate deesterification to degrade the sulfonylurea herbicides by rupturing the ester bond in the herbicides [18]. The *sulE* gene was further cloned and was successfully expressed in *Saccharomyces cerevisiae* [18]. Recently, Sulfometuron-degrading enzyme E3, a type of hydrolase that degrades nicosulfuron by breaking the urea bridge, was obtained from a strain of *Oceanisphaera psychrotolerans*, which was expressed in *Escherichia coli* [19].

The third generation of high-throughput DNA sequencing technology has enabled direct sequencing of the gDNA of *K. pneumoniae* 2N3 with significant savings and increased accuracy [20,21,22]. Currently, the third generation sequencing platforms mainly include the true single molecular sequencing (tSMS) of the Helicos biosciences, the PacBio of Pacific Biosciences, and the nanopore single-molecule technology of Oxford Nanopore Technologies [20,21,22]. In this study, the whole genome of *K. pneumoniae* 2N3 was sequenced and assembled using the PacBio high-throughput sequencing technology. The basic characteristics of the 2N3 genome were further analyzed, and the functional genes were annotated by using databases of the non-redundant (NR) protein, Gene Ontology (GO), Clusters of Orthologous Groups (COGs) proteins, and the Kyoto Encyclopedia of Genes and Genomes (KEGG). The functional genes related to the degradation of chlorimuron-ethyl were (1) preliminarily predicted by using sequence information and functional annotation of genes and (2) further experimentally confirmed by gene knockout technology. These results provide a solid foundation for further exploring the degradation mechanisms of chlorimuron-ethyl by *K. pneumoniae* 2N3.

## 2. Results and Discussion

### 2.1. Genome Sequencing Characteristics

**Figure 1:**
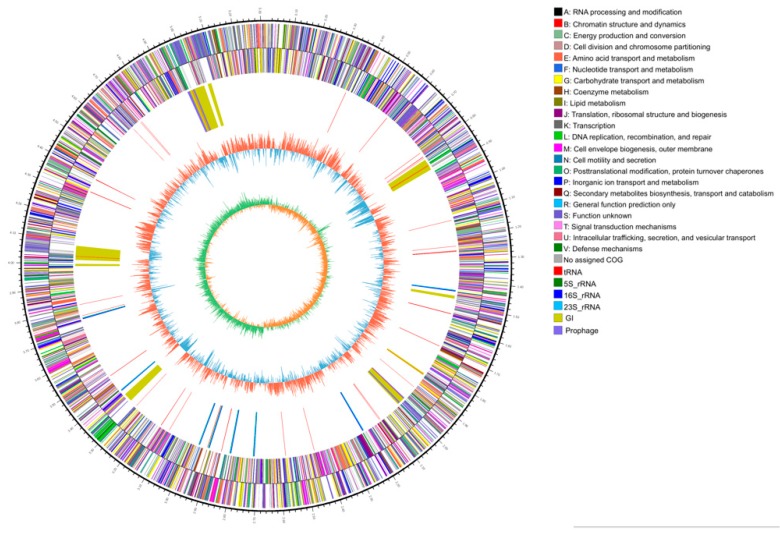
Genome map of *K. pneumoniae* 2N3. The five layers in order from the outmost to the innermost represent: (1) genome indicated by lengths of DNA sequences in kb, (2) and (3) coding sequences (CDS) on the positive strand and negative strand, respectively, with color bars representing clusters of orthologous group (COG) classifications, (4) locations of rRNA and tRNA genes, (5) GC contents with red bars and blue bars proportionally indicating areas with GC contents higher and lower than the average GC content of the entire genome, respectively, and (6) values of GC-skew as calculated using the algorithm of G − C/G + C.

A total of 26,930 reads (183,081,518 bp) corresponding to 348.9 fold genome coverage were obtained. The shortest and the longest sequences were 35 bp and 34,393 bp, respectively, with an average sequence length of 6798 bp. One single contig was obtained by the assembly, and the results showed that the total length of the circular 2N3 genome was 5,319,547 bp with a GC content of 57.33% without undetermined nucleotide bases. The circle map of the 2N3 genome was drawn using Circos (Figure 1). The genome sequence of strain 2N3 was deposited in GenBank with an accession number CP025541 (Bioproject accession: PRJNA427082) under the name of *K. pneumoniae*, previously recognized as *K. jilinsis* [10].

### 2.2. Molecular Characteristics of the 2N3 Genome

**Figure 2:**
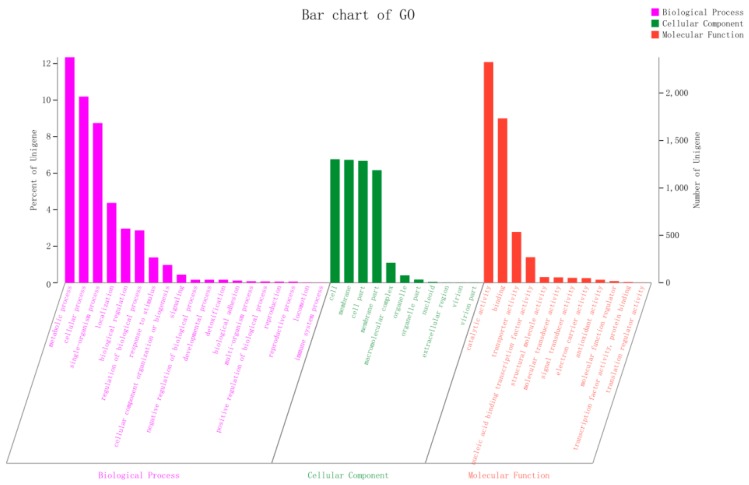
Content distribution of functional genes from *K. pneumoniae* 2N3 based on the Gene Ontology (GO) database.

A total of 5156 coding gene open reading frames (ORFs), ranging from 114 to 6342 bp in length with an average of 906 bp, were predicted in the 2N3 genome with a total length of 4,669,044 bp and a GC content of 58.54% (Table 1). We noted that the number of genes predicted in this genome was similar to those reported in many of the genomes of *Klebsiella* at NCBI (https://www.ncbi.nlm.nih.gov/). A total of 112 non-coding RNAs were also predicted in the 2N3 genome. Furthermore, these 5156 coding genes in the 2N3 genome were annotated by the NR database, while the gene ontology (GO) annotated a total of 287 out of the 5156 ORFs into 3995 GO terms (Figure 2). The highest number of functional genes (2378) were predicted in the group of metabolic process in the domain of biological process, followed by the catalytic activity (2327) in the domain of molecular function, while less than 50 genes were predicted in many GO terms (Figure 2).

**Figure 3:**
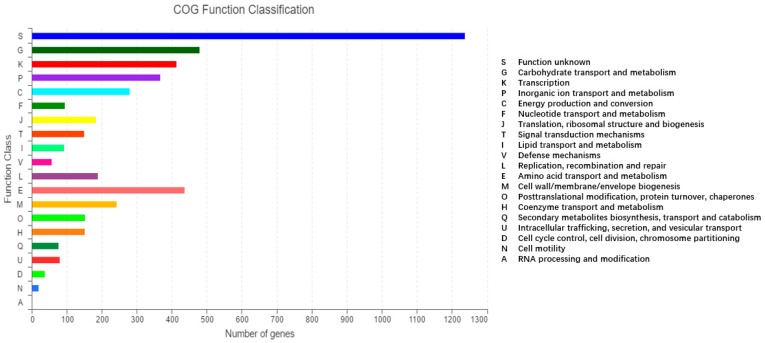
Functional classifications of genes from *K. pneumoniae* 2N3 based on the Clusters of Orthologous Groups (COG) proteins database.

A total of 4648 out of the 5156 ORFs were annotated into 22 categories in the COG database (Figure 3). The COG annotations identified a large amount of genes (1237) with unknown functions, while a total of six groups, each with over 200 genes, were annotated in energy production and conversion (280), amino acid transport and metabolism (437), carbohydrate transport and metabolism (479), transcription (413), cell wall/membrane/envelope biogenesis (243), and inorganic ion transport and metabolism (367) (Figure 3). The least number of genes were annotated in the categories of RNA processing and modification (1) and cell motility (19).

**Figure 4:**
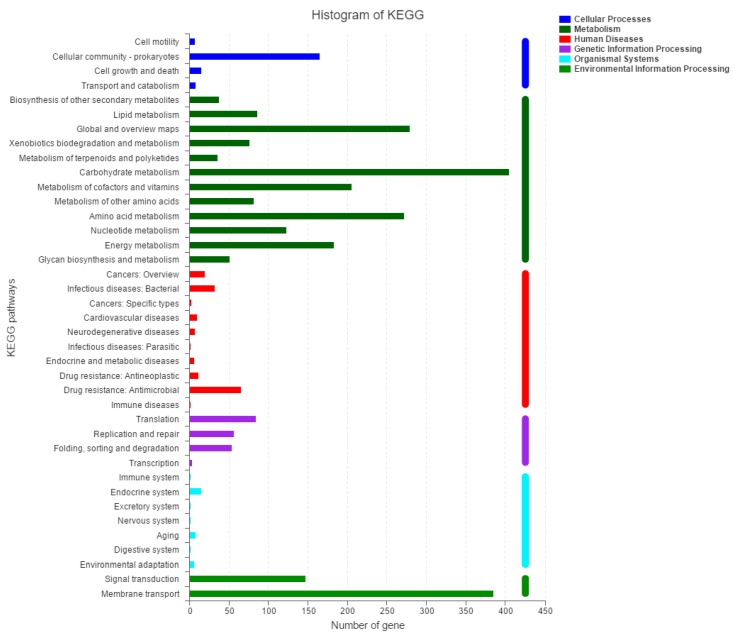
Categories of metabolic pathways of genes from *K. pneumoniae* 2N3 based on the Kyoto Encyclopedia of Genes and Genomes (KEGG) database.

Furthermore, a total of 2960 out of the 5156 ORFs were annotated into a total of 40 biological pathways in the KEGG database (Figure 4). The largest number of genes were annotated into the pathways of carbohydrate metabolism (405) and membrane transport (385). Among the six groups of pathways in the KEGG, the organismal systems contained the least number of genes (37). In the KEGG database (Figure 4). A total of 16, 7, and 451 genes were annotated to have the functions of P450, flavin monooxygenase (FMO), and hydrolase, respectively.

None of the 16 cytochrome P450 coding genes predicted in the 2N3 genome showed the same conserved domain (i.e., CypX belonging to the P-450 family) as compared with the two cytochrome P450 enzymes P450SU1 and P450SU2 present in *Streptomyces griseolus* [14,15], which are responsible for the degradation of sulfonylurea herbicides. Furthermore, the local BLAST (Basic Local Alignment Search Tool) analysis did not identify any matching sequences. Two of the six encoding genes of flavin monooxygenase (ORFs 2804 and 4234) were highly homologous to the nicosulfuron-degrading enzyme FMO. Multiple alignment of DNA sequences using MEGA6 [23] indicated high homology between ORF 4234 and FMO. Three ORFs (4234, 3547, and 2804) and FMO each contain the Pyr_redox_2 superfamily domain (i.e., pyridine nucleotide-disulfide oxidoreductase superfamily). Both ORFs 0934 and 0492 contain the Esterase_713_like domain, which is involved in breaking the ester bond on the halogenated ring by esterase SulE.

### 2.3. Protein Sequence and Phylogenetic Analyses of E3

**Figure 5:**
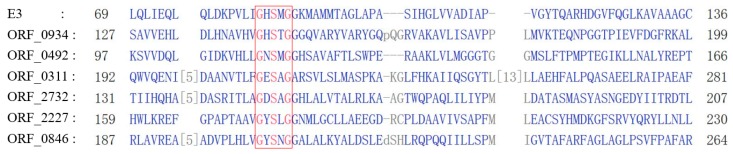
The conserved pentapeptide sequences (as highlighted in the red box) of E3 and six open reading frames (ORFs) from *K. pneumoniae* 2N3 (ORFs 2732, 2227, 0934, 0846, 0492, and 0311). The numbers at both ends of the amino acid sequences indicate the positions of the amino acids in the sequences, and the numbers in brackets indicate the number of amino acids omitted in the alignments.

**Figure 6:**
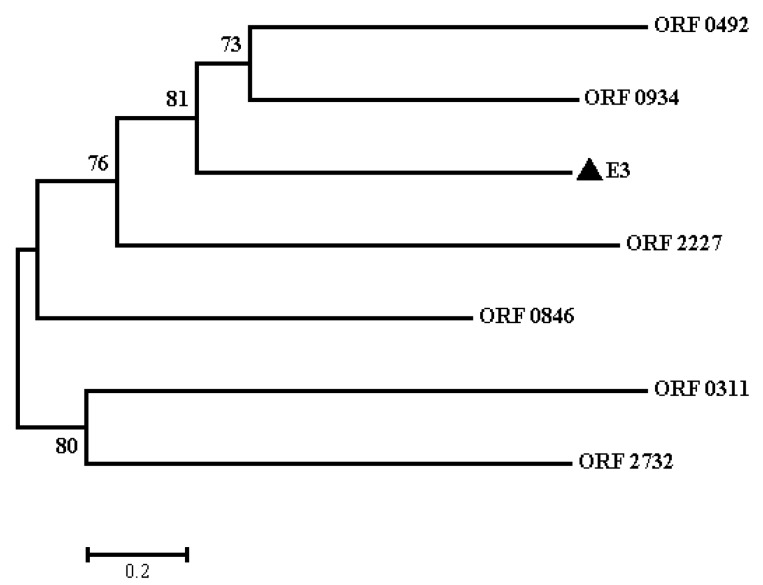
A phylogenetic tree using the neighbor-joining method based on the complete amino acid sequences of E3 (indicated with the symbol of a black triangle) and six ORFs from *Klebsiella pneumoniae* 2N3 (ORFs 2732, 2227, 0934, 0846, 0492, and 0311). Bootstrap values (given as 1–100%) derived from 1000 replicates are given next to the branches. The scale bar refers to the number of amino acid substitutions per site.

Nicosulfuron-degrading hydrolase E3 contains the MhpC (pimeloyl-ACP methyl esterase) and Abhydrolase 1 family domains, the basic sequence (i.e., Gly-X-Ser-X-Gly) of hydrolases, and the catalytic triad structure Ser-Glu-His (Figure 5), functioning to break the carbonyl group in the ester bond [24]. In the 2N3 genome, three ORFs (0934, 0492, and 0166) of the seven hydrolases having relatively high homology with E3 contain both MhpC and Abhydrolase 1 domains. Six ORFs (2732, 2227, 0934, 0846, 0492, and 0311) contain the conserved pentapeptide motif Gly-X-Ser-X-Gly (Figure 5). Despite the presence of the conserved pentapeptide motif, the six ORFs generally have little overall sequence identity to E3 protein. The overall amino acid similarities between these six ORFs and the E3 protein are 10.42%, 12.40%, 18.73%, 8.67%, 16.90%, and 8.27%, respectively. The phylogenetic tree using the neighbor-joining method derived from these six ORFs and E3 using MEGA6 is presented in Figure 6. Four of these six ORFs (2732, 0934, 0492, and 0311) contain the catalytic triad structure Ser-Glu-His. Furthermore, the amino acid sequences of ORFs 2096 (a non-heme chloroperoxidase) and 0492 (a 2-hydroxy-6-oxonona-2,4-dienedioate hydrolase) also contain similar domains as SulE. The phylogenetic tree shows that ORFs 0934 and 0492 are closely related to E3. Therefore, it is speculated that ORFs 0934 and 0492 may be involved in the degradation of chlorimuron-ethyl as well. Furthermore, the wide distribution of these six ORFs in other common species of *Klebsiella*, such as *K. aerogenes*, *K. michiganensis*, *K. oxytoca*, *K. pneumoniae*, *K. quasipneumoniae*, and *K. variicola*, indicates high conservation and potential functions within these bacteria. Currently, it is not known whether these enzymes present in other rare species of *Klebisella*.

### 2.4. Gene Knockout and Degradation of Chlorimuron-ethyl

**Figure 7:**
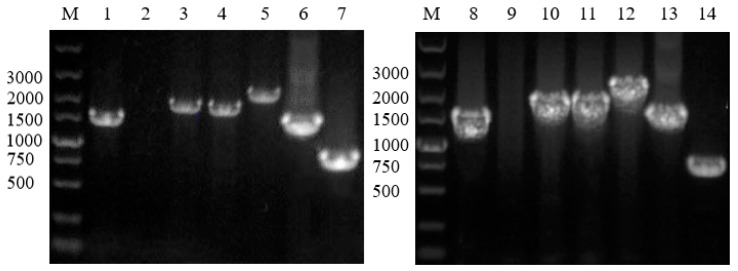
ORFs 0934 and 0492 gene knockout construction examined by PCR. Lane M represents the molecular markers, Lane 1 indicates the amplification of the sequence of the kanamycin resistance gene in ΔORF 0934, Lane 2 the ORF 0934 fragment in ΔORF 0934 (no band), Lane 3 the sequence of the kanamycin resistance gene in ΔORF 0934 + upstream homologous arm, Lane 4 the sequence of the kanamycin resistance gene in ΔORF 0934 + downstream homologous arm, Lane 5 the full length of the kanamycin resistance gene cassette of ΔORF 0934, Lane 6 the control of the kanamycin resistance gene (with the pKD4 plasmid as the template), Lane 7 the ORF 0934 fragment in the wild type (WT), Lane 8 the amplification of the sequence of the kanamycin resistance gene in ΔORF 0492, Lane 9 the ORF 0492 fragment in ΔORF 0492 (no band), Lane 10 the sequence of the kanamycin resistance gene in ΔORF 0492 + upstream homologous arm, Lane 11 the sequence of the kanamycin resistance gene in ΔORF 0492 + downstream homologous arm, Lane 12 the full length of the kanamycin resistance gene cassette of ΔORF 0492, Lane 13 the control of the kanamycin resistance gene (with the pKD4 plasmid as the template), and Lane 14 the ORF 0492 fragment in the wild type (WT).

**Figure 8:**
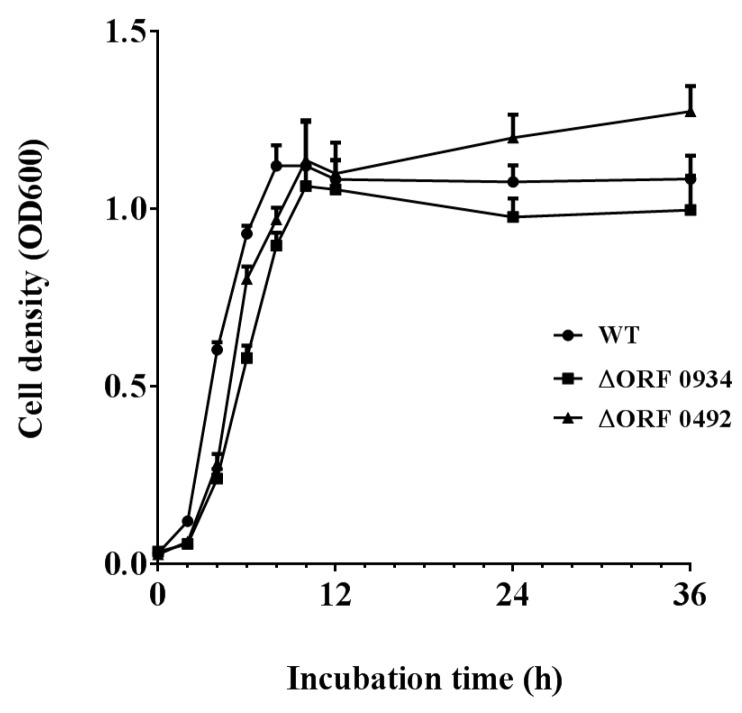
Bacterial growth curves of strains WT, ΔORF 0934, and ΔORF 0492. The error bar indicates one standard deviation.

**Figure 9:**
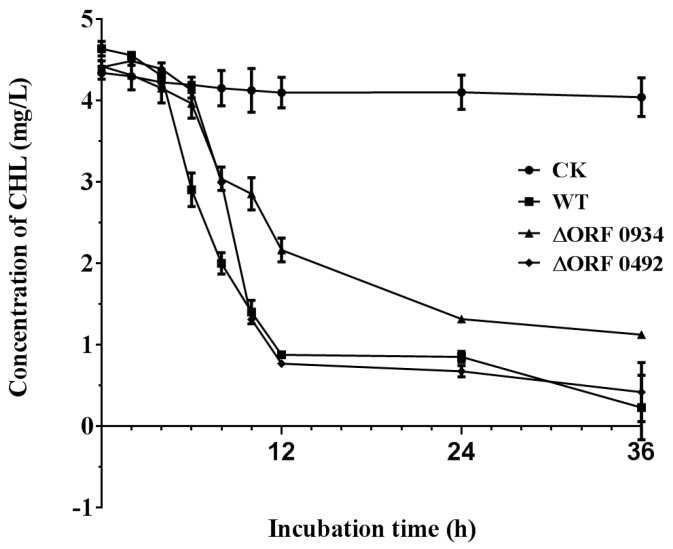
Degradation rates of chlorimuron-ethyl by strains WT, ΔORF 0934, and ΔORF 0492. CK: controls without bacteria. The error bar indicates one standard deviation.

The construction of the gene knockouts of ORF 0934 and 0492 were confirmed by PCR (Figure 7). All three strains (wild type (WT), ΔORF 0934, and ΔORF 0492) grew rapidly on the basal medium containing chlorimuron-ethyl (5 mg/L) in the first 8–10 h in culture. They maintained a similar growth rate for the next 24 h and reached their highest concentrations with ΔORF 0934 being the lowest (OD_600_ = 1.06) and ΔORF 0492 the highest (OD_600_ = 1.14) (Figure 8). These results revealed similar growth patterns between the WT and the two knockouts (ΔORF 0934 and ΔORF 0492) during the entire course of the experiment. These three strains degraded the chlorimuron-ethyl rapidly in 8 h in culture and reached their highest degradation rates at 36 h, with ΔORF 0934 being the lowest at 74.56% and WT the highest at 95.06%, respectively (Figure 9). The degradation rates of chlorimuron-ethyl by ΔORF 0934 were slower than those by the WT, and almost no degradation was detected when the concentration of chlorimuron-ethyl reached 1 mg/L (Figure 9). The degradation rates of chlorimuron-ethyl by ΔORF 0492 were similar to those by ΔORF 0934 for the first 8 h but faster after 8 h and comparable to those by the WT. These results indicated that both ΔORF 0934 and ΔORF 0492 showed decreased degradation rates of chlorimuron-ethyl in the first 8 h in culture compared to the WT by 25.60% and 24.74%, respectively, indicating that ORFs 0934 and 0492 are involved in the degradation of chlorimuron-ethyl. The strain ΔORF 0934 showed consistently lower degradation rates on chlorimuron-ethyl than the WT after 8 h, further indicating that strain 2N3 probably produces multiple types of chlorimuron-ethyl degrading enzymes. Possible functional redundancies are speculated since all strains removed about 75% of the chemicals in the time course of the experiment, indicating that these genes are not essential for the degrading process under these laboratory conditions. At present, we cannot rule out the possibility that these two genes are not essential for the degrading process under laboratory conditions. It is possible that even though we knocked out these two genes, some other genes may still have been functioning to encode proteins that were functional along the degradation pathway to cause the decrease of the chemicals. It is also possible that there were multiple enzymes with the same or similar functions of degrading the chemicals.

The degradation of chlorimuron-ethyl by 2N3 is also comparable to the results obtained by other studies. For example, when cultured in a liquid medium at 30–35 °C with a pH value of 6–7, strain 2N3 was able to degrade 83.5% and 92.5% of the chlorimuron-ethyl with the initial concentrations of 20 mg/L and 100 mg/L, respectively [10]. Furthermore, the degradation of chlorimuron-ethyl by *Stenotrophomonas maltophilia* D310-3 [25], *Hansschlegelia* sp. strain CHL1 [26], and *Enterobacter ludwigii* sp. CE-1 [11] reached the highest rates of 89.9%, 95%, and 90% cultured under optimal conditions in 6, 4, and 7 days, respectively.

## 3. Materials and Methods

### 3.1. Bacterial Strain

*Klebsiella pneumoniae* 2N3 was isolated by enrichment culturing from the sludge taken from an industrial wastewater treatment tank (Shenyang Chemical Industry Research Institute, Shenyang, China) and was stored at the China Center for Type Culture Collection (accession number CCTCC NO: M 209248). Bacteria of strain 2N3 cultured at −80 °C and preserved in glycerol were incubated on Luria-Bertani (LB) plates at 30 °C overnight. Single colonies were picked the next day and cultured in a liquid LB medium at 30 °C and 200 rpm to the logarithmic phase. The bacterial cells were collected by centrifugation to extract genomic DNA using the Tiangen blood/tissue/cell genomic DNA extraction kit (Tiangen Biotech, Beijing, China).

### 3.2. Genome Sequencing, Assembly, and Annotation

The genome of 2N3 was sequenced using the third-generation DNA sequencing platform PacBio RSII SMRT [27]. Due to the sequencing principles deployed by the PacBio system (i.e., each DNA molecule was sequenced multiple times) and the high genomic coverage obtained, a second sequencing method, i.e., Illumina MiSeq, was applied to verify the DNA base bias. Reads were assembled using the Hierarchical Genome Assembly Process (HGAP) [28] and canu [29] to obtain the final assembly. The errors of the PacBio assembly were corrected using the Illumina reads with Pilon [30]. Functional genes were predicted using Glimmer gene-finding [31], transfer RNAs were detected with tRNAscan-SE [32] using default parameters, and ribosomal RNA was identified by RNAmmer [33]. The coding genes were annotated by BLAST analysis using GenBank’s Non-Redundant (NR) protein database at NCBI (http://www.ncbi.nlm.nih.gov/). The functions of the genes were annotated by the Gene Ontology (GO) database [34], the metabolic pathways were annotated by the Kyoto Encyclopedia of Genes and Genomes (KEGG) database [35], and protein-coding genes were annotated by the Clusters of Orthologous Groups (COG) proteins database [36] using BLAST analysis. Gene coding for enzymes, including cytochrome P450 (P450SU1 and P450SU2), flavin monooxygenase (FMO), and hydrolases (e.g., SulE and E3), which are responsible for the degradation of sulfonylurea herbicides, were predicted by the local BLAST analyses (BioEdit version 7.0.5.2) using reported protein sequences of the sulfonylurea degrading enzymes in order to compare them with all of the predicted genomic genes in 2N3. The genes with high homology (36.1% between E3 and ORF 0934 and 38.8% between E3 and ORF 0492) identified by the local BLAST analysis were further examined using the Conserved Domain Search Service (CD Search) tool at NCBI (http://www.ncbi.nlm.nih.gov/Structure/cdd/wrpsb.cgi) to predict the conserved structural domains.

### 3.3. Protein Structural and Phylogenetic Analyses

The conserved pentapeptide motif Gly-X-Ser-X-Gly of nicosulfuron-degrading hydrolase E3 and predicted ORFs from *Klebsiella pneumoniae* 2N3 (ORFs 2732, 2227, 0934, 0846, 0492, and 0311) were aligned using the constraint-based multiple alignment tool (COBALT) at NCBI (https://www.ncbi.nlm.nih.gov/tools/cobalt/cobalt.cgi?LINK_LOC=BlastHomeLink). MEGA6 was used to construct phylogenetic trees using the neighbor-joining method to generate bootstrap values on the phylogenetic trees based on 1000 replicates [23].

### 3.4. Gene Knockout and Degradation of Chlorimuron-Ethyl

**Figure 10:**
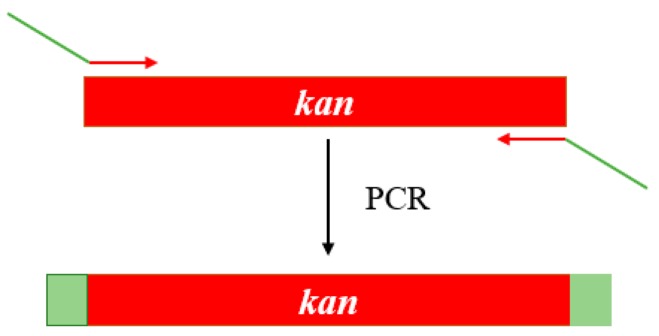
The construction of the kanamycin resistance gene cassette. *Kan* indicates the kanamycin resistance gene in red zones, while the green areas indicate the intergenic regions. The arrows indicate the locations of primers for PCR.

To further provide the experimental evidence to support the annotated functions of ORFs 0934 and 0492, both genes were knocked out using the Lambda–Red system (Shanghai North Connaught bio technology Co., Ltd., Shanghai, China). The construction of the gene knockouts was confirmed by PCR. A plasmid pKD4 containing the kanamycin resistance gene was used as the template to amplify the kanamycin resistance gene and to construct the kanamycin resistance gene cassette (Figure 10). The 5′ end of the primers contained 60 bp, which were homologous to either ends of the target genes. (Table 2).

**Figure 11:**
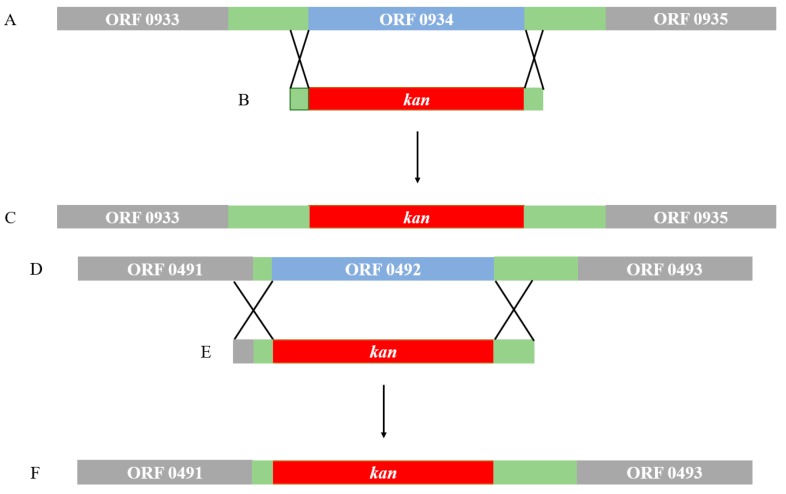
The construction of the two genetic knockouts ΔORF 0934 and ΔORF 0492. A and D show the locations of the target genes (ORFs 0934 and 0492 in blue areas), B and E are the kanamycin resistance gene cassettes showing the 60 bp regions at each end homologous to the target genes to be deleted, and C and F show the genetic structure of the knockouts. The green zones indicate the intergenic regions. Cross symbols between A and B, and D and E indicate the locations of the 60 bp homologous sequences (Table 2). *Kan* indicates the kanamycin resistance gene in red zones. Grey areas indicate the flanking ORFs.

The technique of homologous recombination was used to insert the kanamycin resistance gene cassette into the location of the target gene (Figure 11). These technical strategies ensure that the expressions of the flanking genes around the target genes are not affected.

To determine the effects of the gene knockouts on degradation of chlorimuron-ethyl, both the mutants and the wild type (WT) strains were cultured in the basal medium (containing glucose, 5 g; KH_2_PO_4_, 0.5 g; MgSO_4_·7H_2_O, 0.2 g; K_2_HPO_4_, 0.5 g; NaCl, 0.2 g; NH_4_NO_3_, 1.6 g, and double distilled water, 1000 mL) with chlorimuron-ethyl added (5 mg/L) at 30 °C and 150 rpm. A biophotometer (Eppendorf, city, state abbreviation if any, country) was used to measure the optical density (OD_600_) of the bacterial suspension. The values of OD_600_ were used to estimate the concentration of bacterial population. To put it simply, bacterial growth is positively correlated with the values of OD_600_, and the higher values of OD_600_ indicate the higher concentrations of the bacterial populations. The concentrations of bacterial cells were determined by sampling regularly at 0, 2, 4, 6, 8, 10, 12, 24, and 36 h. No bacteria were added in the controls. The liquid chromatography (HPLC, Shimadzu, LC20A, city, state abbreviation if any, country) with a column C18 was used to measure the concentrations of chlorimuron-ethyl residues at 30 °C at 0, 2, 4, 6, 8, 10, 12, 24, and 36 h. The mobile phase contains methanol, water, and acetic acid (volume of 70, 30, and 0.3, respectively) running at a flow rate of 1 mL/min, with an injection volume of 20 μL and a detection wavelength of 256 nm. The degradation rate of chlorimuron-ethyl was calculated by the formula ((C_0_ − C_t_)/C_0_)∗100%, where C_0_ and C_t_ are the concentrations of chlorimuron-ethyl at time 0 and time t, respectively. Control standard purchased from Sigma-Aldrich (Shanghai, China; Cat # 32874) was used to quantify the chlorimuron-ethyl degradation. All experiments were repeated thrice. The average of three measurements with a standard deviation was calculated to plot the graphs (Figure 8 and Figure 9).

## 4. Conclusions

*Klebsiella* is generally considered as having great potential in its applications in bioremediation because species in this genus are widely distributed in nature, showing strong environmental adaptability and rapid reproductive capacity. The completely sequenced genome of *Klebsiella pneumoniae* 2N3 will further provide insight into the degradation of chlorimuron-ethyl and other sulfonylurea herbicides such as metsulfuron-methyl, tribenuron-methyl, rimsulfuron, ethametsulfuron, and nicosulfuron, and bioremediation to control environmental pollution [10]. To date, there are few studies on the enzymes responsible for the degradation of sulfonylurea herbicides. Most of the reported enzymes are coded by genes having low similarity with the identified genes. Some enzymes (e.g., esterase SulE) have shown degradation activity without cofactors and energy, while other enzymes (e.g., cytochrome P450) require support and regulation by the complex multi-component system in order to degrade the substrates. Therefore, the whole genome of *Klebsiella pneumoniae* 2N3 will not only help us identify more genes for chlorimuron-ethyl degrading enzymes, but also provide scientific support for further exploration of the metabolic pathways and mechanisms regulating these enzymes.

## Figures and Tables

**Table 1:** Molecular characteristics of the genome of *K. pneumoniae* 2N3.

Characteristics	*K. pneumoniae* 2N3
Length (bp)	5,319,547
GC content	57.33%
Ns (%)	0
No. of plasmid	0
No. of coding genes	5156
Total bases (bp)	4,669,044
Length variation (range in bp)	114–6342
Average length (bp)	906
Ns (%)	0
No. of non-coding RNA	112
No. of ribosomal RNA	25
No. of transfer RNA	87

**Table 2:** Primers and sequences used in the construction of the kanamycin resistance gene cassettes. Sequences in lower cases are 60 bp homologous to the portions of the 5′ and 3′ ends of the target genes.

Primers	Sequences
FKF0934-F	5′-gcggcctgataccagacgcggtctggtccaggatcggcgaatcaagctagacagggtaag TGTGTAGGCTGGAGCTG-3′
FKF0934-R	5′-tggcgcccagggcgctggcggaatgccattaacggcactctgcccggcaaagagggcaga ATCCTCCTTAGTTCCTATTCC-3′
FKF0492-F	5′-gatcgccggattcggctcgctgagcgccaccaccgagatttgattaaccgggagactaac TGTGTAGGCTGGAGCTG-3′
FKF0492-R	5′-tgttccgcgtcgcgcagctgccgggccagcgcgtcaagagaaaaagtcatcatttactcc ATCCTCCTTAGTTCCTATTCC-3′
